# Transcriptomic and Metabolic Profiles of Apple Peels of Different Colors

**DOI:** 10.3390/plants14213304

**Published:** 2025-10-29

**Authors:** Pingxing Ao, Yan Ma, Kang Luo, Yumei Ding, Hongjia Zhang, Yue Xu, Shaojie Yuan, Meng Zhang, Hui Guo, Gengyun Li, Yan Zhao, Jianxiang Liu, Leifeng Zhao, Yun Zheng

**Affiliations:** 1 Yunnan International Joint Laboratory of Durian Functional Genomics, College of Landscape and Horticulture, Yunnan Agricultural University, Kunming 650201, China; 2015045@ynau.edu.cn (P.A.); mayan7645@163.com (Y.M.); lk1689252548@163.com (K.L.); zhjzhanghongjia@163.com (H.Z.); xuyue4452@163.com (Y.X.); ysj8955@126.com (S.Y.); 2020054@ynau.edu.cn (M.Z.); ylyjyc@126.com (H.G.); gengyun@fudan.edu.cn (G.L.); 15638583350@163.com (Y.Z.); 2 State Key Laboratory for Conservation and Utilization of Bio Resources in Yunnan, Yunnan Agricultural University, Kunming 650201, China; 3 College of Food Science and Technology, Yunnan Agricultural University, Kunming 650201, China; 2022033@ynau.edu.cn; 4 College of Life Sciences, Zhejiang University, Hangzhou 310027, China; jianxiangliu@zju.edu.cn; 5 College of Big Data, Yunnan Agricultural University, Kunming 650201, China

**Keywords:** apple, peel color, LAR1, catechin, epicatechin, flavonoid pathway, transcriptomic profile, metabolic profile

## Abstract

Apple (*Malus domestica*) is a woody fruit tree belonging to the Rosaceae family, genus *Malus*. It has been widely reported that MYB transcription factors are critical regulators for the red color of apple peel by activating the expression of anthocyanin biogenesis genes. However, it is still not clear what the molecular mechanism for the yellow color of apple peel is. In order to investigate key genes and metabolites responsible for yellow coloration of apple peel, three strains of apples, “Venus Gold (Ype)” with yellow peel, “Yanfu8 (Mpe)” with medium red peel, and “Red love” with dark red peel, were selected in this study. Transcriptomic and metabolomic profiles were obtained for the peels of the three apple strains. After analyzing the transcriptomic profiles and being verified with qRT-PCR experiments, our results suggest that LAR1 is a critical gene for the yellow color of Ype peel. Analysis of metabolomic profiles revealed that the abundances of catechin and epicatechin in Ype peel were higher than those in Rpe, indicating an important reason for the yellow color of Ype peel. Furthermore, when comparing volatile metabolites from Ype, Mpe, and Rpe, hundreds of volatile metabolites show significantly different abundances, suggesting that apples with different peel colors have different odors. Our results uncover a novel metabolic mechanism for yellow coloration, where high expression of LAR1 promotes accumulation of catechin and epicatechin, providing the first integrated transcriptomic and metabolomic evidence for this pathway in apple peel.

## 1. Introduction

Apple (*Malus domestica*) is a plant of the genus *Malus* of the Rosaceae family [1]. Apples are an important fruit crop grown in temperate zones of the world. As the most immediate visual quality attribute, apple peel color is a primary determinant of consumer preference and directly influences market acceptance and commercial value. Fruit color is an important factor in consumer preference, and fruit coloration is one of the most important agronomic traits affecting apple fruit quality [2]. Red-fleshed apples have received extensive attention from apple breeders and consumers because of their attractive colors and high functional nutrient contents [3]. The color of the apple peel is determined by the content of anthocyanins, carotenoids, and chlorophylls and their distribution on the peel surface.

Anthocyanins are water-soluble, widely distributed secondary metabolites that play an important role in epidermal pigmentation in many plant organs and microorganisms. Their spectral properties often result in red, blue, and purple coloration of different plant organs (flowers, fruits, and other plant tissues), due to the accumulation of anthocyanins in the pulp and peel of fruits [4,5,6,7]. The six common types of anthocyanins widely present in nature are pelargonidin chloride, cyanidin chloride, peonidin chloride, delphinidin chloride, malvidin chloride, and petunidin chloride [8,9]. Furthermore, these compounds have received considerable attention owing to their inherent nutritional benefits, distinctive pharmacokinetic characteristics, elucidated pharmacological actions, and capacity to enhance health [10,11].

Anthocyanidin synthesis, accumulation pathways, and related gene expression have been extensively studied in many plants. The anthocyanidin biosynthesis pathway belongs to a specific branch of the flavonoid biosynthesis pathway, and its synthesis process is co-regulated by two classes of genes, i.e., structural genes and transcription factors [12,13]. The relevant structural genes include PAL, CHS, CHI, F3H, DFR, LDOX, and UFGT, which are synergistically expressed during apple fruit development, with a significant positive correlation between their transcript levels and anthocyanin concentration [14,15]. The synthetic substrate of anthocyanidin is phenylalanine, which is synthesized by phenylalanine ammonia-lyase (PAL), cinnamate 4-hydroxylase (C4H), 4-coumarate coenzyme A ligase (4CL), and 4-coumarate CoA ligase (4CL). The formation of 4-coumaroyl-CoA is catalyzed by cinnamate 4-hydroxylase (C4H) and 4-coumarate coenzyme A ligase (4CL), followed by the reaction of 4-coumaroyl-CoA with malonyl-CoA catalyzed by chalcone synthase (CHS) to produce yellow-colored chalcone, which is converted to colorless chalcone by chalcone isomerase (CHI). Flavanone (Naringenin). In the anthocyanidin synthesis pathway, naringenin is catalyzed by flavanone 3-hydroxylase (F3H) to form dihydrosorbinol. Later, leucocyanidin was synthesized by dihydroflavonol-4-reductase (DFR) catalysis, which in turn undergoes the action of anthocyanidin synthase (ANS) to form anthocyanidins [16,17,18,19,20]. Three genes encoding flavonoid 3’-hydroxylase (F3’H) have been identified in apples, and the transcript levels of the *MdF3’H* gene, as well as other anthocyanin biosynthesis genes, are higher in red-skinned varieties than in yellow-skinned varieties throughout apple fruit development [21].

The second category includes transcription factors that can influence the intensity and pattern of anthocyanin accumulation and broadly control the expression of many different biosynthetic genes [22]. It has been shown that MYB transcription factors are involved in anthocyanin synthesis in apple peel. *MdMYB1* and *MdMYB10* are two key transcription factors that activate the biosynthesis of anthocyanin in apple [23,24,25]. Over-expression of *MdMYB9* and *MdMYB11* promoted accumulation of anthocyanin but also proanthocyanidin (PA) accumulation in apple calluses [26]. Under jasmonate (JA) treatment, *MdMYB9* and *MdMYB11* are induced to enhance the accumulation of anthocyanin and PA [26]. On the other side, two other MYBs, *MdMYB6* and *MdMYB17*, repress biogenesis of anthocyanin in apple by competing with *MdMYB1* and *MdMYB10* [27,28]. The WD40 protein (*MdTTG1*) of apple was found to promote the accumulation of anthocyanin glycosides, and anthocyanins combine with sugar to form stable anthocyanidins, which are mainly stored in vesicles in apple fruits [29].

The anthocyanin content plays an important role in the degree of coloration of red apples, and the content and distribution pattern of anthocyanins in the peel lead to different fruit color phenotypes. Beyond their role in pigmentation, these flavonoids are potent antioxidants, and their consumption is associated with various health benefits, meaning that peel color can serve as a visual indicator of the fruit’s nutritional potential [29,30]. Cyanidin-3-galactoside is the most abundant in apple peels, accounting for approximately 80% of the total anthocyanin content [31]. Although it is known that anthocyanin is important for the red color of apple peels, the molecular mechanism of the yellow color of apple peel is largely unknown. While the MYB-mediated activation of anthocyanin biosynthesis is a well-documented mechanism for red pigmentation in apple peel, the metabolic and transcriptional basis for yellow coloration remains elusive. It is particularly unclear whether the yellow phenotype simply arises from the absence of anthocyanins or is an active process involving the biosynthesis and accumulation of specific yellow flavonoids, such as flavonols or catechins. This fundamental gap in our understanding limits the comprehensive mapping of the flavonoid pathway in apple and constrains targeted breeding efforts for yellow-skinned varieties.

Therefore, elucidating the genetic and metabolic basis of peel coloration is not only of fundamental scientific interest but also of paramount importance for molecular breeding strategies aimed at developing novel apple varieties with enhanced visual appeal and nutritional profiles. In this study, we selected three varieties of apples, namely “Venus Gold” with yellow peel, “YanFu8” with medium red peel, and “Red Love” with dark red peel, to investigate the molecular mechanism of the differences in apple peel color. The peels of three selected apple cultivars show very different gene expression patterns and metabolite patterns. By examining the transcriptomic profiles and being verified with qRT-PCR experiments, our results suggest that LAR1 is a key gene that contributes to the yellow color of Ype. By analyzing metabolic profiles of the same samples, our results suggest that cyanidin 3-O-glucoside is the key metabolite that contributes to the red color of apple peel, and catechin and epicatechin are the key metabolites that are critical for the yellow color of apple peel in the selected apple cultivars. Interestingly, our results show that the volatile metabolites of peels of the three cultivars of apples also demonstrated significant differences, suggesting that apples with different peel colors also had different odors. These results provide new insights into the coloration of apple peels, which is of great value in the breeding of apples.

## 2. Results

### 2.1. Morphological Observations on Different Cultivars of Apples

**Figure 1:**
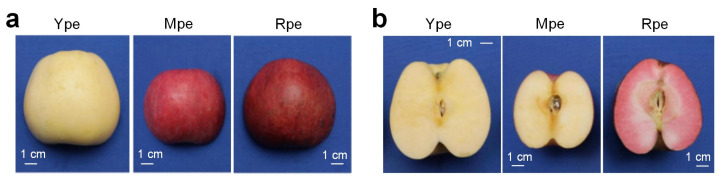
**Peels and fleshes of the three selected cultivars of apples.** (**a**) Mature peels of the three selected cultivars of apples. (**b**) Mature flesh of the three selected cultivars of apples.

According to National Standard for Fresh Apples of China (GB/T-10651-2008) [32], ripe fruits have characteristics unique to their cultivars. Different cultivars of apples have different skin colors during ripening, and the three cultivars in this study fulfilled the color requirements of mature fruits. As shown in Figure 1, the Ype variety has a yellow fruit surface, fruit with fragrance, no sour taste, and golden-yellow flesh. Mpe belongs to the Fuji family, with more than 50% of its fruit surface covered in red and yellowish flesh. Rpe is a full-red variety, with more than 90% of its fruit surface covered in red, and its flesh is red with a sour taste. All three types of apples have excellent cosmetic qualities and are attractive to consumers.

### 2.2. Analysis of the Transcriptomic Profiles of Apple Peels in Different Colors

**Figure 2:**
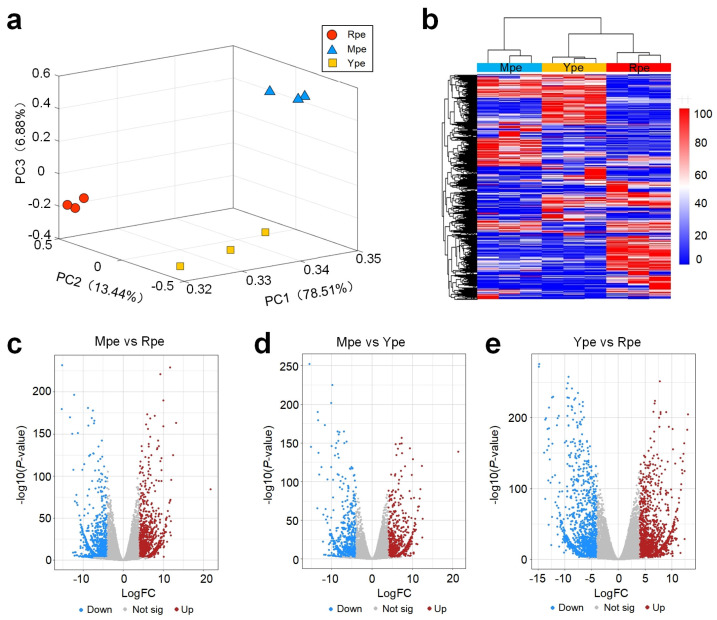
**Analysis of gene expression pattern in apple peels of different colors.** (**a**) Principal component analysis using the gene expression profiles. (**b**) Hierarchical clustering using the gene expression profiles. (**c**–**e**) DEGs in the comparisons of Mpe vs. Rpe, Mpe vs. Ype, and Ype vs. Rpe, respectively. In the volcano plots, logFC ≥ 4 and p≤ 0.001 were used to call DEGs. The source data of Part (**a**,**b**) are available in Table S29. The source data of Part (**c**–**e**) were available in Tables S30–S32.

RNA-Seq profiles were generated for three biological replicates of Ype, Mpe, and Rpe, respectively. Next, gene expression levels were calculated from the obtained RNA-Seq profiles (see Materials and Methods section). We then performed PCA and clustering analysis using the gene expression data. As shown in Figure 2a, the results of PCA showed that the three groups of samples were widely separated, and the individual samples were closely clustered within each group, indicating that the selected samples in different groups had very different gene expression patterns. The small intra-group differences, especially for Mpe and Ype (Figure 2a), indicate good reproducibility of the samples within the same groups. Clustering analysis showed that samples of the same groups clustered together, which was very similar to that of PCA, indicating that there were large differences in gene expression between different groups of samples (Figure 2b).

### 2.3. DEGs and the Expression of Important Genes in Apple Peels with Different Colors

The three groups of samples were compared to identify differentially expressed genes (DEGs) with edgeR [33] (see Materials and Methods for details). When comparing Rpe to Ype, 1503 and 1692 genes were significantly upregulated and downregulated in Rpe, respectively (Figure 2c, Tables S3 and S4, respectively). In the comparison between Rpe and Mpe, there were 2291 DEGs, of which 985 were upregulated and 1306 were downregulated in Mpe, respectively (see Figure 2d, Tables S5 and S6, respectively). When comparing Mpe to Ype, 697 and 967 genes were significantly upregulated and downregulated in Mpe, respectively (Figure 2e, Tables S7 and S8, respectively). In summary, these results suggest that there are large differences in gene expression for apple peels with different colors.

**Figure 3:**
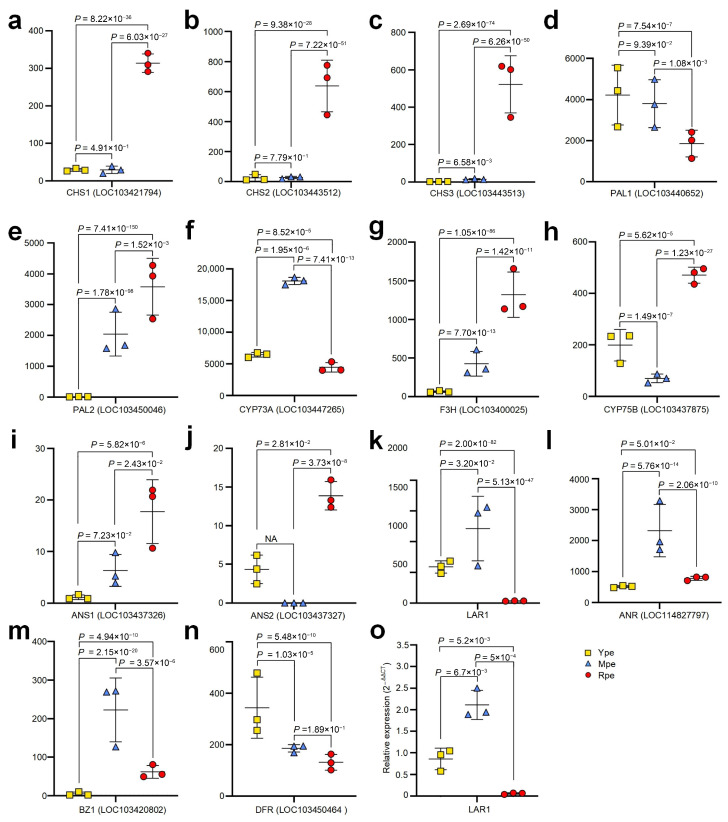
**Comparisons of expression levels of important genes in flavonoid biosynthesis pathway.** Multiple test-corrected *p*-values were based on edgeR. (**a**) CHS1 (LOC103421794). (**b**) CHS2 (LOC103443512). (**c**) CHS3 (LOC103443513). (**d**) PAL1 (LOC103440652). (**e**) PAL2 (LOC103450046). (**f**) CYP73A (LOC103447265, i.e., C4H). (**g**) F3H (LOC103400025). (**h**) CYP75B (LOC103437875, i.e., C3H). (**i**) ANS1 (LOC103437326). (**j**) ANS2 (LOC103437327). (**k**) LAR1. (**l**) ANR (LOC114827797). (**m**) BZ1 (LOC103420802). (**n**) DFR (LOC103450464). (**o**) The relative expression of LAR1. The source data are available in Table S33.

We then examined known genes that are related to anthocyanin biosynthesis. As shown in Figure 3a–c, three CHS genes, i.e., CHS1 (LOC103421794), CHS2 (LOC103443512), and CHS3 (LOC103443513), showed significantly higher expression in Rpe than in both Ype and Mpe. PAL1 (LOC103440652) had significantly higher expression in Ype and Mpe than in Rpe (Figure 3d). However, PAL2 (LOC103450046) showed a reversed expression pattern compared to PAL1, with the highest expression in Rpe, followed by Mpe, and the lowest expression in Ype (Figure 3e). CYP73A (LOC103447265, i.e., C4H) had the highest expression in Mpe, followed by Ype, and the lowest expression in Rpe (Figure 3f).

The expression of F3H (LOC103400025) was the highest in Rpe, followed by Mpe, and the lowest in Ype (Figure 3g). CYP75B (LOC103437875, i.e., C3H) also had the highest expression in Rpe, next in Ype, and the lowest expression in Mpe (Figure 3h). Two ANS genes, ANS1 (LOC103437326) and ANS2 (LOC103437327), had the highest expression in Rpe (Figure 3i and Figure 3j, respectively). ANS1 had higher expression in Mpe than in Ype (Figure 3i), but ANS2 had higher expression in Ype than in Mpe (Figure 3j).

LAR1, ANR (LOC114827797), and BZ1 (LOC103420802) had the highest expression in Mpe among the three groups (Figure 3k, Figure 3l and Figure 3m, respectively). LAR1 had significantly higher expression in Ype than in Rpe (Figure 3k). BZ1 had significantly higher expression in Rpe than in Ype (Figure 3m). DFR1 (LOC103450464) was significantly upregulated in Ype compared to Mpe and Rpe (Figure 3n).

We also validated the expression of LAR1 with qRT-PCR. As shown in Figure 3o, LAR1 was significantly upregulated in Ype than Rpe, which was similar to the results of RNA-Seq profiles (see Figure 3k).

### 2.4. Potential Functions of DEGs in the Coloration of Apple Peels

In order to investigate the potential functions of DEGs in apple peels with different colors, KEGG pathway enrichment analysis was performed. The 1503 genes upregulated in Rpe when compared to Ype (Table S3) were significantly enriched in 15 pathways (corrected *p* < 0.05, Table S9), including glutathione metabolism, phenylpropanoid biosynthesis, and flavonoid biosynthesis. The 1692 genes downregulated in Rpe when compared to Ype (Table S4) were significantly enriched in four pathways (corrected *p* < 0.05, Table S10), including photosynthesis and photosynthesis antenna proteins.

When comparing Mpe and Rpe, 985 genes upregulated in Mpe were only significantly enriched in phenylpropanoid biosynthesis (corrected *p* < 0.05, Table S11). And 1306 genes downregulated in Mpe were mainly enriched in 7 KEGG pathways, including taurine and hypotaurine metabolism, butanoate metabolism, and flavonoid biosynthesis (corrected *p* < 0.05, Table S12).

In the comparisons of Mpe vs. Ype, 697 genes upregulated in Mpe were significantly enriched in seven KEGG pathways, including sesquiterpenoid and triterpenoid biosynthesis and isoquinoline alkaloid biosynthesis (corrected *p* < 0.05, Table S13). Meanwhile, 967 genes downregulated in Mpe were enriched in 14 KEGG pathways, including cyanoamino acid metabolism, phenylpropanoid biosynthesis, and glutathione metabolism (corrected *p* < 0.05, Table S14).

In summary, when comparing Rpe to both Ype and Mpe, the genes upregulated in Rpe were significantly enriched in flavonoid biosynthesis (Table S9 and Table S12, respectively). These results suggest that flavonoid biosynthesis is enhanced in Rpe samples.

### 2.5. Analysis of Soluble Metabolic Profiles of Apple Peels in Different Colors

The metabolic profiles of apple peel samples of Ype, Mpe, and Rpe were analyzed based on the MetWare Database (MWDB), a self-constructed database of extensively targeted metabolites. A total of 808 metabolites were detected in the peel samples of the three apple cultivars (Table S12).

**Figure 4:**
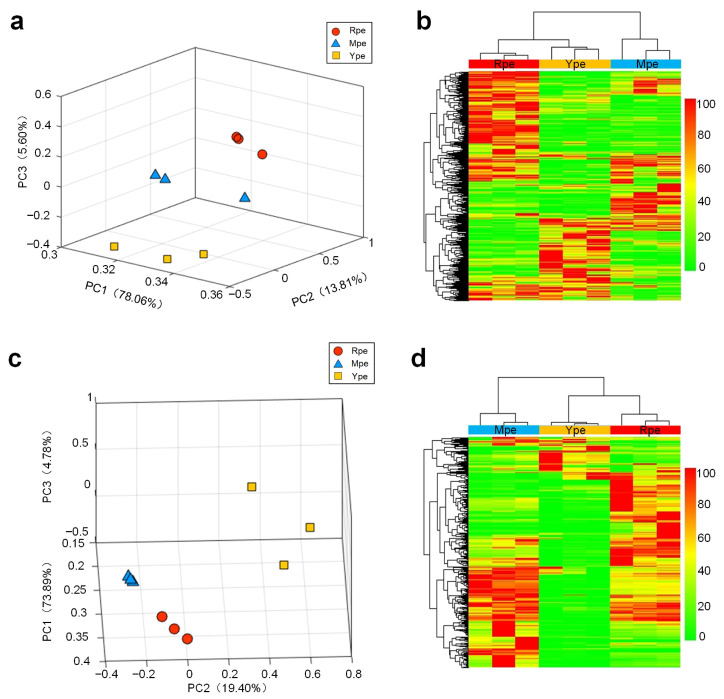
**Metabolite analysis of apple peel samples with different colors.** (**a**) PCA using the soluble metabolic profiles of apple peel samples. (**b**) Hierarchical clustering using the soluble metabolic profiles of apple peel samples. (**c**) PCA using the volatile metabolic profiles of apple peel samples. (**d**) Hierarchical clustering using the volatile metabolic profiles of apple peel samples. The source data of Part (**a**,**b**) are available in Table S34. The source data of Part (**c**,**d**) are available in Table S35.

In the PCA using the abundances of soluble metabolites (Figure 4a), it can be seen that Rpe and Ype are farther apart, and the three samples within each group are relatively clustered. In Figure 4b, the results of the clustering were consistent with the PCA, with the Mpe, Rpe, and Ype samples clustered individually in a cluster. These results indicate that Ype, Mpe, and Rpe have different metabolite abundance patterns. In addition, the results of the PCA and clustering analyses using the metabolic profiles were also consistent with the results of the transcriptome analysis in Figure 2. This indicates that Ype, Mpe, and Rpe samples show different gene expression patterns, which might contribute to their different metabolite abundance patterns.

### 2.6. Enriched Pathways of Soluble Metabolites with Different Abundances

In the following, we examined the enriched KEGG pathways for soluble metabolites with different abundances in Ype, Mpe, and Rpe. As shown in Figure S1a, upregulated metabolites in Ype compared to Rpe were enriched in aminoacyl-tRNA biosynthesis (ko00970), 2-oxocarboxylic acid metabolism (ko01210), glycine, serine, and threonine metabolism (ko00260), and cysteine and methionine metabolism (ko00270). In comparison, upregulated metabolites in Ype were enriched in flavone and flavonol biosynthesis (ko00944), phenylpropanoid biosynthesis (ko00940), galactose metabolism (ko00052), flavonoid biosynthesis (ko00941), and lysine degradation (ko00310) (see Figure S1b), suggesting more active anthocyanin biosynthesis in Rpe than in Ype.

When comparing Mpe to Ype, as shown in Figure S1c, upregulated metabolites in Mpe compared to Ype were enriched in flavone and flavonol biosynthesis (ko00944), flavonoid biosynthesis (ko00941), and tropane, piperidine, and pyridine alkaloid biosynthesis (ko00960), suggesting anthocyanin biosynthesis is also very active in Mpe. As shown in Figure S1d, upregulated metabolites in Ype compared to Mpe were enriched in purine metabolism (ko00230) and glyoxylate and dicarboxylate metabolism (ko00630).

As shown in Figure S1e, it is interesting that upregulated metabolites in Mpe compared to Rpe were also enriched in flavone and flavonol biosynthesis (ko00944) and flavonoid biosynthesis (ko00941), suggesting anthocyanin biosynthesis is even more active in Mpe than in Rpe. Phenylpropanoid biosynthesis (ko00940) and pyrimidine metabolism (ko00240) were enriched in the metabolites upregulated in Rpe compared to Mpe (see Figure S1f).

Notably, among the metabolites upregulated in Ype, we identified significantly higher abundances of catechin and epicatechin than in Rpe (Table S17). These compounds are the fundamental building blocks (flavan-3-ol monomers) for the biosynthesis of proanthocyanidins (PAs, also known as condensed tannins). The enrichment of the flavonoid biosynthesis pathway, coupled with the specific accumulation of these precursors in Ype, suggests a redirected metabolic flux towards the PA branch in the yellow peel. Crucially, catechin and epicatechin are known to be sensitive to light, particularly UV and blue light [34,35], which can induce their oxidation into yellowish products. Therefore, the concerted higher abundance of catechin and epicatechin, driven by an active biosynthetic pathway, provides a compelling biochemical rationale for the yellow coloration of the Ype peel.

### 2.7. Analysis of Volatile Metabolites

Volatile organic compounds are often subdivided into several classes, including terpenoids, alkanes, aromatic hydrocarbons, esters, and aldehyde. In this study, 589 volatile metabolites were detected (Table S16). Specifically, 247, 336, and 370 volatile metabolites with different abundances were identified in the three comparisons of Mpe vs. Rpe, Mpe vs. Ype, and Ype vs. Rpe, respectively (Tables S23 to S28, respectively). In the comparison between Ype and Rpe, 18 metabolites were downregulated in Rpe (Table S23). And a total of 352 metabolites were upregulated in Rpe, with terpenoids having the highest proportion of all metabolites, followed by esters, hydrocarbons, and alcohols being the most abundant (as shown in Table S24). Several terpenoids are significantly upregulated in Rpe when compared to Ype, such as 4-(1,5-dimethyl-1,4-hexadienyl)-1-methyl-Cyclohexene (XMW0975) and 1-methyl-4-(1,2,2-trimethylcyclopentyl)cyclohexa-1,3-diene (D338) (see Table S24). Terpenoids constituted the most prominent class, with key compounds such as farnesene—a sesquiterpene known for its contribution to the characteristic “green”, “woody” notes in apple aroma and implicated in superficial scald development—being highly enriched in Rpe. Additionally, esters, which are pivotal for the fruity aroma of apples, were markedly more abundant in Rpe (Table S24). Specifically, we observed significantly higher levels of esters like hexyl acetate and butyl acetate, which are renowned for imparting sweet, fruity, and pear-like aromas and are hallmark volatiles in cultivars.

In PCA and clustering analysis, the samples of the three groups were clustered together and different groups were well separated (Figure 4c). Similarly, in the clustering analysis, samples of Ype, Mpe, and Rpe were also clustered together (Figure 4d). These results suggest that the three apple cultivars have very different odors and that the biochemical pathways underlying volatile biosynthesis are differentially regulated in peels of different colors. The heightened abundance of terpenoids in Rpe may be linked to an upregulation of the terpenoid backbone biosynthesis pathway, while the ester enrichment points to altered activity in the lipoxygenase (LOX) pathway and alcohol acyltransferases (AATs). Consequently, the Rpe cultivar’s volatile signature, rich in specific terpenes and esters, likely contributes to a more intense and complex aroma profile compared to the Ype and Mpe cultivars, directly linking peel color to a distinguishable olfactory trait.

### 2.8. Joint Analysis of the Transcriptomic and Metabolic Profiles

**Figure 5:**
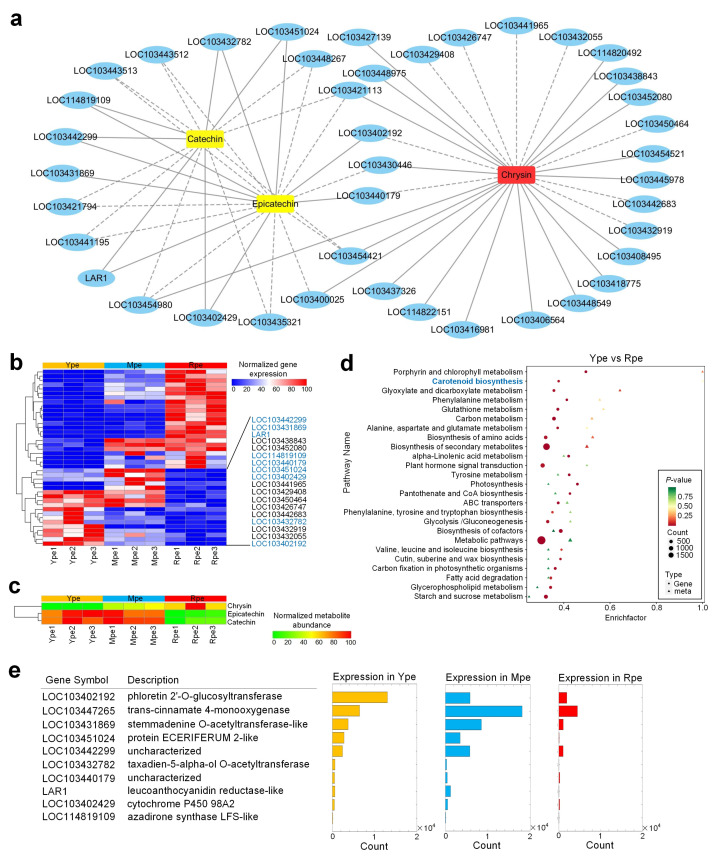
**Joint analysis of transcriptomic and metabolic profiles when comparing Ype to Rpe.** (**a**) The correlation network of genes and metabolites when comparing Ype and Rpe. Blue ovals represent genes, yellow rectangles represent metabolites that are more abundant in Ype than in Rpe, and red rectangles represent metabolites that are more abundant in Rpe than in Ype. Solid and dashed lines represent positive and negative correlation coefficients between genes and metabolites, respectively. (**b**) The expression levels of genes in Part (**a**). The nine genes in blue symbols have positive correlation coefficient values with catechin and/or epicatechin. (**c**) The abundances of metabolites in Part (**a**). (**d**) The details of the nine genes that show significant positive correlation coefficient values with catechin and/or epicatechin. LOC103447265 is also shown because this gene has a significant positive correlation coefficient with catechin (MWSHY0167) in the comparison between Mpe and Rpe (see Figure S3a and Table S42). The source data of Part (**a**) are available in Table S36. The source data of Part (**b**–**e**) are available in Tables S37, S38, and S39, respectively.

When analyzing a correlation network of genes and metabolites showed different abundances between Ype and Rpe, we found that chrysin, epicatechin, and catechin showed very high correlation values with 41 genes (Figure 5a). Among them, epicatechin and catechin were upregulated in Ype, whereas chrysin was upregulated in Rpe (Figure 5a,c). As shown in Figure 5a, nine genes had positive correlation values between their expression levels and abundances of epicatechin and catechin. These nine genes showed higher expression levels in Ype than in Rpe (Figure 5b). These suggest that these nine genes are involved in the more active biosynthesis or accumulation of epicatechin and catechin in Ype than in Rpe. On the other side, chrysin had higher abundances in Rpe (Figure 5c) and showed positive correlation with many genes with higher expression in Rpe (Figure 5b).

In the combined enrichment analysis of genes and metabolites, carotenoid biosynthesis is enriched in the DEGs and metabolites when comparing Ype to Rpe (Figure 5d). When comparing Mpe to Rpe, the DEGs and metabolites were enriched in biotin metabolism and phorphyrin, chlorophyll metabolism, and many other pathways (Figure S2a). When comparing Mpe to Ype, the DEGs and metabolites were enriched in phorphyrin and chlorophyll metabolism, nitrogen metabolism, and many other pathways (Figure S2b).

When comparing Mpe to Rpe, epicatechin and catechin were also more abundant in Mpe than in Rpe (Figure 5c), and they had significant positive correlation values with 10 genes (Figure S3a). Nine of these ten genes also showed positive correlation values with epicatechin and catechin in the correlation network of Ype vs. Rpe (Figure 5a,b). In the correlation network between Mpe and Rpe (Figure S3b), all of the seven metabolites were more abundant in Rpe. Five of the seven metabolites are flavonoids, suggesting an enhanced flavonoid biosynthesis pathway in Rpe.

As shown in Figure 5e, we carefully examined the 10 genes that have positive correlation coefficient values with epicatechin and catechin in Figure 5a and Figure S3a. We found these genes were generally highly expressed in Ype, particularly LOC103402192 (phloretin 2^′^-o-glucosyltransferase, UGTF1).

### 2.9. Phylogenetic Analysis of MYB Transcription Factors

In order to identify the relationship between MYB transcription factors in apple, Arabidopsis, and peach, seven genes that had been reported to be functional in anthocyanin synthesis were selected. Then, a phylogenetic tree was constructed using protein sequences of four genes of other species with reported functions in anthocyanin biosynthesis and 442 MYBs of apple. As shown in Figure S4a, the MYB genes that played roles in anthocyanin synthesis in apple and other species (with red names in Figure S4a) were clustered in Branch 1, while no genes were found in Branches 2 to 6.

*MdMYB1* and *MdMYB10* are two key transcription factors that activate the biosynthesis of anthocyanin in apple [23,24,25,36,37,38]. In our transcriptomic profiles, *MdMYB1* and *MdMYB10* showed similar expression in the three groups of apple peels, with the highest expression in Rpe, then lower expression in Mpe, and the lowest in Ype (Figures S5a and S5b, respectively). Methyl jasmonate (MeJA) induces *MdMYB9* and *MdMYB11*, whose over-expression promotes the accumulation of anthocyanin and proanthocyanidin accumulation [26]. *MdMYB9* and *MdMYB11* directly regulate ANS, ANR, and LAR, which are key enzymes in the biosynthesis pathway of anthocyanin [26]. In our RNA-Seq profiles, *MdMYB9* showed the highest expression in Rpe, lower expression in Mpe, and the lowest expression in Ype (Figure S5c). This suggests that *MdMYB9* might contribute to the accumulation of anthocyanin and proanthocyanidin in Rpe. *MdMYB6a* and *MdMYB17* repress biosynthesis of anthocyanin in apple by competing with *MdMYB1* and *MdMYB10* [27,28]. In our results, the expression of *MdMYB6a* (LOC103401412) did not change severely in Ype and Rpe (Figure S5d), suggesting this gene is non-functional in regulating the biosynthesis of anthocyanin in the apple cultivars selected. However, *MdMYB6b* (XP_028950860.1) had much higher expression in Rpe compared to Ype (Figure S5e), suggesting that this gene might be involved in the biosynthesis of anthocyanin in Ype samples. *MdMYB11* almost did not express in Ype and Rpe, and showed higher expression in Mpe than in both Ype and Rpe (Figure S5f), suggesting that *MdMYB11* plays a role in anthocyanin accumulation in Mpe. *MdMYB110*, a paralog of *MdMYB10*, upregulates anthocyanin biosynthesis in tobacco (*Nicotiana tabacum*) [39]. In our RNA-Seq profiles, *MdMYB110* had slightly higher expression in Mpe but did not show significant different expression in Ype and Rpe (Figure S5g). Furthermore, *MdTTG1* (LOC103406603) had the highest expression in Rpe, then lower in Mpe, and the lowest expression in Ype (Figure S5h).

We had a close look at other MYBs that were closely clustered with MYBs that were involved in biosynthesis of anthocyanin, as shown in Figure S4b–e. We then examined the expression levels of the genes in Figure S4b–e, and found that at least 10 additional MYBs showed significantly different expression levels in Ype, Mpe, and Rpe. As shown in Figure S6, three MYBs were upregulated in Rpe compared to Ype (Figure S6a,c,d), and 7 of these MYBs were upregulated in Ype compared to Rpe (Figure S6b,e–j). Since they showed very close phylogenetic distances to MYBs involved in the biosynthesis of anthocyanin and showed significantly different expression levels in Ype and Rpe, these 10 genes might play roles in the coloration of apples. However, more studies are needed to further verify their functions.

### 2.10. Genes and Metabolites Involved in the Flavonoid Biosynthetic Pathway

**Figure 6:**
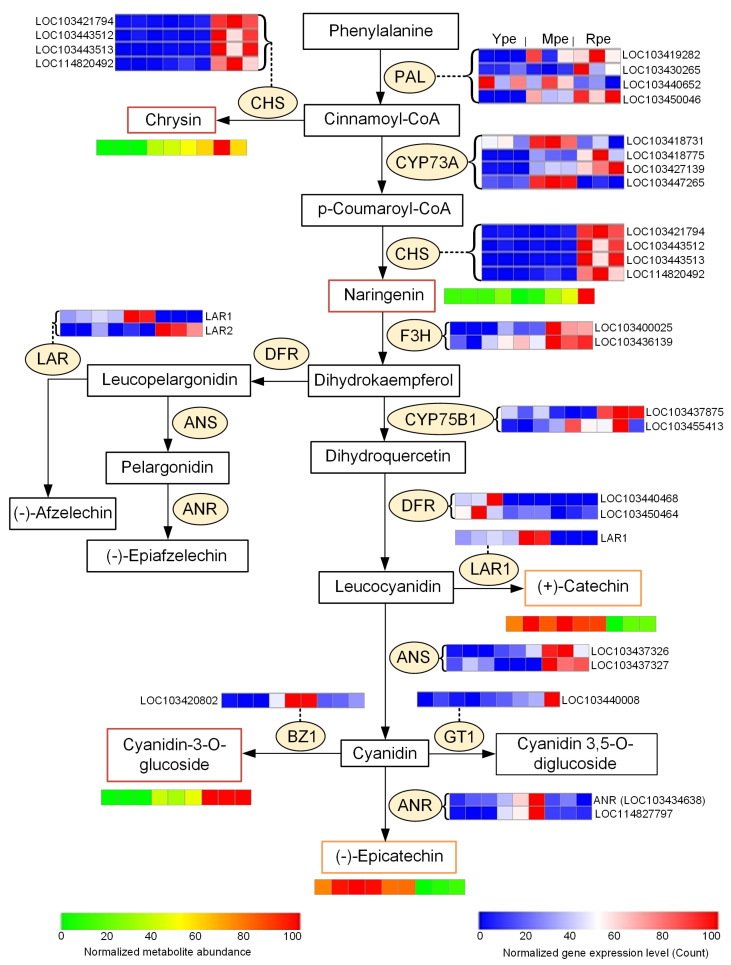
**A schematic view of the flavonoid biosynthesis pathway.** The flavonoid biosynthesis pathway was revised from the flavonoid biosynthesis pathway of KEGG. Rectangles represent metabolites. Yellow and red rectangles represent metabolites that are more abundant in Ype and Rpe, respectively. Ovals represent enzymes (genes) in the biosynthesis pathway. The expression levels of the genes and the abundances of metabolites in Ype, Mpe, and Rpe were shown beside them. The source data are available in Table S40.

To analyze the effects of genes on the flavonoid biosynthetic pathway in the apple peels with different colors, we examined the expression patterns of structural genes related to the biosynthetic pathway. As shown in Figure 6, two PAL genes (LOC103430265, LOC103450046) had higher expression inside the Rpe, suggesting their positive roles in accumulation of anthocyanin in Rpe and Mpe. Another PAL gene (LOC103440652) had the highest abundance in Ype.

One CYP73A (LOC103447265) was highly expressed within the Mpe (Figure 5e). Four CHS were highly expressed in Rpe, especially LOC103421794, LOC103443512, and LOC103443513, which was consistent with the fact that the metabolites Chrysin and Naringenin were the most abundant in Rpe (as shown in Figure 6). One F3H (LOC103400025) and one CYP75B1 gene (LOC103437875) are upregulated in Rpe, suggesting that they played a role in the accumulation of anthocyanin in Rpe.

A DFR (LOC103450464) gene had higher expression in Ype than in Rpe, which might contribute to higher abundance of leucocyanidin in Ype than in Rpe. In the following step, LAR1 was upregulated in Ype when compared to Rpe, which is consistent with the fact that catechin had higher abundance in Ype than in Rpe. Therefore, the two consecutive enlargements by DFR and LAR1 might contribute to the higher abundance of catechin in Ype than in Rpe. Catechin was also more abundant in Mpe than in Rpe.

The BZ1 gene was the most highly expressed in Mpe, and the corresponding metabolite anthocyanin (cyanidin-3-o-glucoside) gradually increased from Ype to Rpe, reaching its highest abundance in Rpe. Two ANS genes (LOC10343726 and LOC10343727) showed higher expression in Rpe than in Ype. One ANR (LOC114827797) had the highest abundance in Mpe, with epicatechin being the most abundant in Ype, then moderately abundant in Mpe, and the least abundant in Rpe.

In summary, DFR (LOC103450464) and LAR1, with higher expression in Ype than in Rpe, contribute to higher abundance of catechin in Ype than in Rpe. Epicatechins have significantly higher abundances in Ype than in Rpe, suggesting their roles in the yellowish color of Ype. Chrysin, naringenin, and cyanidin-3-o-glucoside, had higher abundances in Rpe than in Ype, contributing to the red color of Rpe.

## 3. Discussion

Many studies showed a positive correlation between anthocyanin accumulation in the peel and one or more structural genes in the anthocyanin synthesis pathway [40]. Our results suggest that CHS1, CHS2, CHS3, PAL2, F3H, CYP75B, ANS1, and ANS2 with higher expression levels in Rpe contribute to the red color of Rpe. Interestingly, our results showed that leucoanthocyanidin reductase 1 (LAR1) had a higher expression level in Ype than in Rpe (see Figure 3k,o). VvLARs produce (+)-catechin from leucocyanidin in grapevine [41]. Recombinant VvLAR1 and VvLAR2 convert 4β-(S-cysteinyl)-catechin and 4β-(S-cysteinyl)-epicatechin into (+)-catechin and (-)-epicatechin in vitro, respectively [41]. Thus, LAR1 with higher expression in Ype is supposed to produce higher abundances of catechin and epicatechin in Ype than in Rpe.

More than 700 different pigments have been identified in plants from 27 families and 73 genera [42]. However, exploration of the mechanism of apple peel color development is still limited. High contents of anthocyanins and anthocyanin derivatives potentially contribute to the bright red color of fruit varieties [43]. In this study, metabolomic analysis revealed that flavonoids were the most abundant substances in the peel of the three apple varieties, with a total of eight flavonoids identified. More specifically, in Ype with yellow color, the anthocyanin content is lower than in Mpe and Rpe. In contrast, in Mpe and Rpe, cyanidin-3-o-glucoside is the main form of anthocyanin and has the highest level in the Rpe variety. Cyanidin-3-O-glucoside has been reported to be the major anthocyanin in apples and red pears [44], and both also belong to the Rosaceae family, suggesting that closely related plants from the same family may contain similar anthocyanin species. In summary, cyanidin-3-o-glucoside is the main anthocyanin that contributes to the red color in the Rpe and Mpe selected in this study.

Currently, research on apple skin coloration remains focused on the formation mechanism of red pigments. However, yellow apples appearing in the market are gaining increasing popularity. Modern science knows very little about the formation mechanism of yellow pigments in yellow apples, which limits the development of new yellow apple varieties. In this study, we integrated transcriptomic and metabolomic data to investigate the key factors influencing yellow pigment formation in apples. As shown in Figure 6, catechin and epicatechin have higher abundances in the Ype with yellow peel than in Rpe. Catechin and epicatechin are sensitive to UV and blue light, which can cause them to form yellowish products [34,35]. Different catechin monomers in fresh tea leaves undergo distinct color changes following enzymatic oxidation [45]. A high proportion of epicatechin leads to the formation of more yellow oxidation products [46]. Therefore, our results suggest that higher abundances of catechin and epicatechin in Ype than in Rpe and Mpe contribute to the yellow peel color of Ype.

The flavor of apples is influenced by a variety of factors, with volatile metabolites being particularly important. In addition to the distinct coloration, our data also indicate that the different peel colors are associated with unique volatile profiles, suggesting a potential co-regulation of color and aroma traits. More than 350 volatile aroma substances have been identified in apple fruits, and most of the volatiles are esters, alcohols, aldehydes, theophyllenes, and ketones [47,48]. Ethyl 2-methyl butyrate, 2-methylbutyl acetate, and hexyl acetate have been attributed to the aroma of Fuji apples [49]. The main compounds responsible for the aroma of “Ning Qiu” and “Golden Delicious” are olefins and esters [50,51]. Furthermore, it has been shown that methyl butyrate is important for strawberry aroma [52,53].

In this study, analysis of the volatile metabolome revealed that most volatile metabolites were more abundant in Rpe, especially terpenoids, than in Mpe and Ype. Our results also indicate that the volatile metabolites in Rpe, Mpe, and Ype have very different abundance patterns, suggesting their different odors. Geranyl isobutyrate is characterized by its sweet, floral, and rose-like aroma with distinct fruity notes. We detected significantly elevated expression of geranyl isobutyrate and butyl caprylate in the Rpe compared to Ype (Table S24), which is a key factor explaining why RPE-type apples exhibit the characteristic apple aroma [54]. Research has increasingly identified geranyl isobutyrate as a key differentiator for certain apple varieties. For instance, its presence and concentration have been correlated with the complex, floral top-notes found in cultivars such as “Pink Lady” and “Gala” [49]. It acts as a “fingerprint” compound, adding a layer of sophisticated, almost tropical nuance that distinguishes these varieties from those with simpler, greener aromatic profiles. Studies using gas chromatography–olfactometry have confirmed geranyl isobutyrate as a key aroma-active compound in several modern cultivars, underscoring its role beyond mere presence to active sensory contribution [55]. The role of butyl caprylate is now understood to extend beyond a simple volatile. Its waxy character is sensorially associated with the perception of ripeness and a full-bodied, almost creamy mouthfeel [56]. Furthermore, some esters were markedly more abundant in Rpe than in Ype (Table S24), suggesting that Rpe released a sweet, fruity, and pear-like odor.

TFs perform pivotal roles in the regulation of color changes and in the pigment biosynthetic pathway [57]. *MdMYB1* is the key transcription factor that activates other genes in the anthocyanin biogenesis pathway [23]. Two structural genes, *MdDFR* and *MdUFGT*, in the flavonol synthesis are activated by *MdMYB1* [23]. *MdMYB1* and *MYB10* also transcriptionally activate *MdVHA-B1* and *MdVHA-B2*, which control anthocyanin accumulation [25]. In our RNA-Seq profiles, *MdMYB1* has the highest expression in Rpe, then moderate expression in Mpe, and the lowest expression in Ype (Figure S5a). *MdMYB10* positively regulates anthocyanin biogenesis in apple flesh and peel [24]. Our results showed that *MdMYB1*, *MdMYB10*, and *MdMYB110* showed higher expression in Rpe than in Ype (see Figure S5a, S5b, and S5g, respectively), which is consistent with their roles in the anthocyanin biogenesis and contributes to the red color of Rpe. The WD40 protein (*MdTTG1*) of apple was found to promote the accumulation of anthocyanin [29]. Our results showed that *MdTTG1*, with higher expression in Rpe than in Ype, also played a role in the red coloration of Rpe.

The apple is a globally renowned, high-quality fruit. Through years of hybridization and artificial domestication, a rich variety of apple cultivars has emerged. Significant genetic differences exist among different apple varieties, resulting in distinct colors and flavors [58,59]. This study selected three apple cultivars with distinct fruit skin colors, i.e., “Venus Gold (Ype)”, “Yanfu8 (Mpe)”, and “Red Love (Rep)”, as the experimental subjects. The discovery of high expression of catechin and epicatechin is one of the key factors in the formation of yellow fruit skin. However, it still requires further investigation to examine whether this regulatory mechanism also applies to the formation of yellow coloration in other apple cultivars.

## 4. Materials and Methods

### 4.1. Experimental Materials and Treatments

Three apple cultivars were examined in this study, i.e., the “Red Love” with red peel and red flesh (Rpe), the “YanFu8” with red peel and yellow flesh (Mpe), and the “Venus Gold” with yellow peel and yellow flesh (Ype). The apple trees of three selected cultivars were grown in Xiaolong Village, Malong County, Qujing, Yunnan, China, which had an altitude of 2030 m, an average annual temperature of 13.8 °C, and an annual rainfall of 1020 mm. The fruit samples of the three apple cultivars were picked on 205 days after flowering (24 October 2021, 9:00–10:00 A.M.). The peels were immediately frozen in liquid nitrogen and stored in a −80 °C refrigerator. The peel samples of this study were collected on the same day and during the same time period. Three biological replicates were collected for each of the three cultivars.

### 4.2. Preparing the Sequencing Libraries and Sequencing

Total RNA was extracted, and RNA and its concentration were measured using a Qubit 2.0 fluorometer (Thermo Fisher, Waltham, MA, USA). RNA integrity was accurately detected by the Agilent 2100 Bioanalyzer (Agilent, Santa Clara, CA, USA). The presence of DNA contamination was analyzed using agarose gel electrophoresis. RNA-Seq libraries were then constructed when RNA integrity was ensured. The qualities of the libraries were tested, and preliminary quantification was performed using Qubit 2.0 (Thermo Fisher, Waltham, MA, USA). The obtained RNA-Seq libraries were pooled and sequenced on an Illumina Novaseq 6000 sequencer (Illumina, San Diego, CA, USA). RNA-Seq profiles were generated for three biological replicates for the three selected cultivars, i.e., Ype, Mpe, and Rpe, respectively. In total, we obtained over 45 million reads with a sequencing mode of 2 × 150 nt for each of the nine apple peel samples (Table S1). A total of 49.09 GB of clean data were obtained for the nine apple peel samples, with an average sequencing error rate of 0.03%. These demonstrated a high quality and reliability of the transcriptomic profiles obtained (Figure S1 and Table S1). The nine RNA-Seq profiles were available at the NCBI GEO Database with the series accession number GSE304477.

### 4.3. Processing of the RNA-Seq Profiles

The genome sequence and gene annotation of apple (cultivar: Golden Delicious) were downloaded from the NCBI Genome database under the accession number GCA_002114115.1. The obtained RNA-Seq profiles were aligned to the apple genome (cultivar: Golden Delicious) using the Burrows–Wheeler algorithm (v0.7.17-r1188) [60] with the option of “-t 24”. The average mapping ratio was 98.0% (Table S1). Then, the obtained alignments in the SAM format were sorted using SAMTools (v1.12) [61] with the options of “-@ 10-m 50 G”. The transcripts were assembled using Cufflinks (v2.2.1) [62] with the options of “-p 4–library-type fr-firststrand”. The transcripts in different profiles were merged using cuffmerge in the Cufflinks package [62] with the option of “-p 10”. The merged transcripts were then compared to the original gene annotation of apple (cultivar: Golden Delicious) using cuffcompare in the Cufflinks package [62] with the default options. In total, 44,187 transcripts were identified from the obtained RNA-Seq profiles. Finally, as listed in Table S2, the normalized expression levels of apple genes were obtained using cuffquant (with the options of “-u -p 10”) and cuffnorm (with the option of “-p 8”) in the Cufflinks package [62].

### 4.4. Identifying Differentially Expressed Genes

The mean values for three groups of samples were calculated using MS Excel (Microsoft, Redmond, WA, USA). The genes with average expression levels greater than or equal to 5 RPKM in any group of expression data were kept for further analysis. Differentially expressed genes (DEGs) were identified using the edgeR package (v3.42.4) [33]. Then, the criteria of absolute value of logFC ≥ 4 and corrected p< 0.001 were used to define DEGs. These criteria were used to find appropriate numbers of DEGs since the numbers of DEGs grew very fast if slightly looser criteria were employed. The DEGs in the comparisons of Ype vs. Rpe, Mpe vs. Rpe, and Mpe vs. Ype were listed in Tables S3–S8, respectively. Next, the ggplot2 package was used to plot volcano maps. Finally, KEGG Orthology Based Annotation System (KOBAS) software (v3.0) [63] was used to find enriched KEGG pathways for DEGs. The enriched KEGG pathways for the DEGs in the comparisons of Ype vs. Rpe, Mpe vs. Rpe, and Mpe vs. Ype were listed in Tables S9–S14, respectively.

### 4.5. Principal Component Analysis and Clustering Analysis Using Gene Expression Profiles

The gene expression profiles were also used to perform a principal component analysis (PCA) with the pca function in MatLab (v2019) software (MathWorks, Natick, MA, USA). The hclust function in the pheatmap package was used to perform clustering analysis for the gene expression profiles. The pheatmap function in the pheatmap package was used to visualize the result of the clustering analysis.

### 4.6. Identification and Quantification of Soluble Metabolites

The apple peel samples were put into a freeze-dryer (Scientz-100F, Scientz, Ningbo, China) for vacuum freeze-drying and dissolved in 1.2 mL of 70% methanol extract for UPLC-MS/MS analysis. Nexera X2 (Shimadzu, Kyoto, Japan) and 4500 QTRAP (Applied Biosystems, Waltham, MA, USA) were used to perform the UPLC-MS/MS analysis. Substance characterization was performed based on the secondary spectrum information from a self-built database, MWDB (MetWare DataBase, Wuhan Meteville Biotechnology Co., Ltd., Wuhan, Hubei, China). In total, 808 soluble metabolites were detected from the apple peel samples (Table S15). Metabolite quantification was accomplished by analysis using triple quadrupole mass spectrometry in multiple reaction monitoring (MRM) mode. Analyst software (v1.6.3) (Applied Biosystems, Waltham, MA, USA) was used to process the mass spectrometry data for quality control. To perform quality control, the Coefficient of Variation values, i.e., standard deviations divided by means, of peaks were calculated. The curves of CVs for Ype, Mpe, and Rpe were prepared. In the curves of Ype, Mpe, and Rpe, the percentages of peaks with CV values smaller than 0.5 were all greater than 85%, suggesting good repeatabilities and stabilities of the profiles of soluble metabolites.

### 4.7. Identification and Quantification of Volatile Metabolites

Extraction, identification, and quantification of all metabolites were carried out by Wuhan Meteville Biotechnology Co., Ltd. (Wuhan, Hubei, China). For the volatile metabolites, all apple samples were moved out of the −80 °C refrigerator for liquid nitrogen grinding, then NaCl solution and standard solution were added for HS-SPME extraction with SPME Arrow (CTC Analytics AG, Zwingen, Switzerland). Then, 8890-7000D (Agilent, Santa Clara, CA, USA) was used to perform the GC-MS analysis. All the ions to be assayed in each group were detected separately in periods of time corresponding to the order of peaks. If the retention time of the detected peaks was consistent with the standard reference and the selected ions all appeared in the mass spectra of the samples after deduction of the background, the substance was judged to be assayed. In total, 589 volatile metabolites were detected from the apple peel samples (Table S16). The sample downstream mass spectra file was opened using MassHunter quantitative software (v10.0, Agilent, Santa Clara, CA, USA) for quality control. Similar to soluble metabolite profiles, the curves of CVs for Ype, Mpe, and Rpe were prepared. In the curves of Mpe and Rpe, the percentages of peaks with CV values smaller than 0.5 were all larger than 85%. In the CV curve of Ype, the percentage of peaks with CV values smaller than 0.5 was larger than 70%. These results suggest satisfactory repeatabilities and stabilities of the profiles of volatile metabolites.

### 4.8. Analysis of Metabolic Profiles

The Orthogonal Partial Least Squares Discriminant Analysis (OPLS-DA) method [64] was employed to analyze the soluble and volatile metabolic profiles. OPLS-DA decomposes predictor variables into correlated and uncorrelated components relative to the response, enabling the removal of irrelevant information for improved feature selection. For both the 808 soluble and 589 volatile metabolites, OPLS-DA was performed with the OPLSR.Anal function in the MetaboAnalystR package (v1.0.1) in R software, which combined Orthogonal Signal Correction (OSC) [64,65] and OPLS-DA methods. For all of the OPLS-DA models, the $R^{2}Y$ values are close to 1 and $Q^{2}$ values are above 0.95, which indicates that these models are very stable and reliable. Thus, it is feasible to screen the differential metabolites by the Variable Importance in Projection (VIP) values obtained from these OPLS-DA models. In this study, the following criteria were used to filter differential metabolites: (i) the fold change ≥ 2 or ≤0.5 and (ii) VIP ≥ 1. In total, over 300 soluble metabolites were called as having significantly different abundances in the comparisons between different groups of samples, i.e., Ype vs. Rpe, Ype vs. Mpe, and Mpe vs. Rpe (in Tables S17 to S22, respectively). Similarly, over 200 volatile metabolites were regarded as having significantly different abundances in the comparisons between different groups of samples, i.e., Ype vs. Rpe, Ype vs. Mpe, and Mpe vs. Rpe (in Tables S23 to S28, respectively).

The top 50 metabolites with the largest VIP values were used to calculate Pearson’s correlation coefficient values between their abundances to examine potential co-abundance of these metabolites in the peel samples of different cultivars of apples.

We also performed PCA for the abundances of the 808 soluble and 589 volatile metabolites detected in the apple peel samples, respectively. The pca function of MatLab (v2019) software (Mathworks, MA) was used to perform the PCA analysis. Hierarchical clustering analysis for the abundances of the 808 metabolites and 588 volatile metabolites was performed with the hclust function and then visualized with the pheatmap function in the pheatmap package in R, respectively.

### 4.9. Combined Analysis of the Transcriptomic and Metabolic Profiles

The correlation coefficient between genes and metabolites was calculated with MatLab (v2019) software (Mathworks, MA). The pairs of genes and metabolites that had correlation coefficient values smaller than −0.8 or larger than 0.8 were kept for further analysis. Then, the selected pairs of genes and metabolites were visualized with Cytoscape (v3.10.3) [66].

### 4.10. Phylogenetic Analysis of the MYB Transcription Factors

The protein sequences of apple (cultivar: Golden Delicious) were downloaded from NCBI, and were functionally annotated using eggnog-mapper (v4.5) [67] software to screen for MYB transcription factors. We obtained the 440 MYB family members of apple (as listed in Table S44). Then, we collected MYB genes that were reported to play roles in coloration of fruits, i.e., *PpMYB9*, *PpMYB20*, and *PpMYBPA1* of peach [68], *AtMYB75* [69] and *AtMYB123* [68] of *Arabidopsis thaliana*, *MdMYB110* [39] and *MdMYB10* [24] of apple, and downloaded their protein sequences from NCBI (as listed in Table S44). Finally, 440 MYBs of apple, 5 MYBs of other species, and *MdMYB110/MdMYB10* were used to perform multiple sequence alignment on a Linux server using the ClustalW algorithm (v2.1) [70]. The obtained multiple sequence alignment was then used to build a phylogenetic tree with the maximum likelihood algorithm in MEGA (v11.0.13) [71]. The obtained phylogenetic tree was then visualized with iTOL (v7) [72].

### 4.11. Validating Expression of Selected Genes

First, total RNAs were extracted from apple peel samples of Ype, Mpe, and Rpe, respectively, using the TransZol Up Plus RNA Kit (TransGen Biotech, Beijing, China) according to the manufacturer’s instructions. Next, reverse transcription was performed using FastKing gDNA Dispelling RT SuperMix (TIANGEN, Beijing, China) for gene RT-qPCR. Gene qPCR was performed using a two-step method of 2× Realab Green PCR Fast mixture (LABLEAD, Beijing, China). Each gene’s qPCR system contains 7.5 μL Taq SYBR Green qPCR Premix, 1 μL cDNA template, 0.3 μL primers, and 5.9 μL dd H_2_O. The gene qPCR procedure was as follows: initial denaturation for 30 s at 95 °C, followed by 40 cycles of denaturation at 95 °C for 30 s, annealing, and extension at 60 °C for 30 s. Each reaction was performed for three biological replicates. *Malus domestica* actin was used as the single internal control for calculating the relative expression of genes. The $2^{-\Delta \Delta CT}$ method [73] was used to calculate the relative expression. All primers used in this research are listed in Table S47.

## 5. Conclusions

In this study, we examined the transcriptomic and metabolic profiles of three apple cultivars, i.e., Ype with yellow peel, Mpe with medium red peel, and Rpe with dark red peel. Our results indicate that LAR1 shows higher expression levels in Ype than in Rpe, which potentially contributes to higher abundances of catechin and epicatechin in the Ype with yellow peel than in Rpe. These two components are further produced to yellow products by UV or blue light. Therefore, their higher abundances in Ype might represent one of the reasons for the yellow peel of Ype. On the other side, lower accumulation of anthocyanin in Ype than in Rpe also contributes to the yellow coloration of peel in Ype.

## Data Availability

The nine RNA-Seq profiles are available at the NCBI GEO database https://www.ncbi.nlm.nih.gov/geo (accessed on 1 September 2025) under the series accession No. GSE304477.

## Supplementary Materials

The supporting information can be downloaded at https://www.mdpi.com/article/10.3390/plants14213304/s1.

## Abbreviations

The following abbreviations are used in this manuscript:

CV	Coefficient of variation, i.e., standard deviation divided by mean
DEG	Differentially expressed gene
KEGG	Kyoto Encyclopedia of Genes and Genomes
Mpe	Apple cultivar “Yanfu8” with a medium red peel
OPLS-DA	Orthogonal partial least squares discriminant analysis
OSC	Orthogonal signal correction
PCA	Principal component analysis
Rpe	Apple cultivar “Red love” with a dark red peel
VIP	Variable importance in projection
Ype	Apple cultivar “Venus Gold” with a yellow peel
