# Supplementary material for: Transcriptomic and Metabolic Profiles of Apple Peels of Different Colors
Source: Plants (Basel). 2025 Oct 29;14(21):3304. doi: 10.3390/plants14213304 (PMC12610381; doi:10.3390/plants14213304)
Supplement: Supplementary file 1 [file plants-14-03304-s001.zip › Supplementary-figures.pdf]

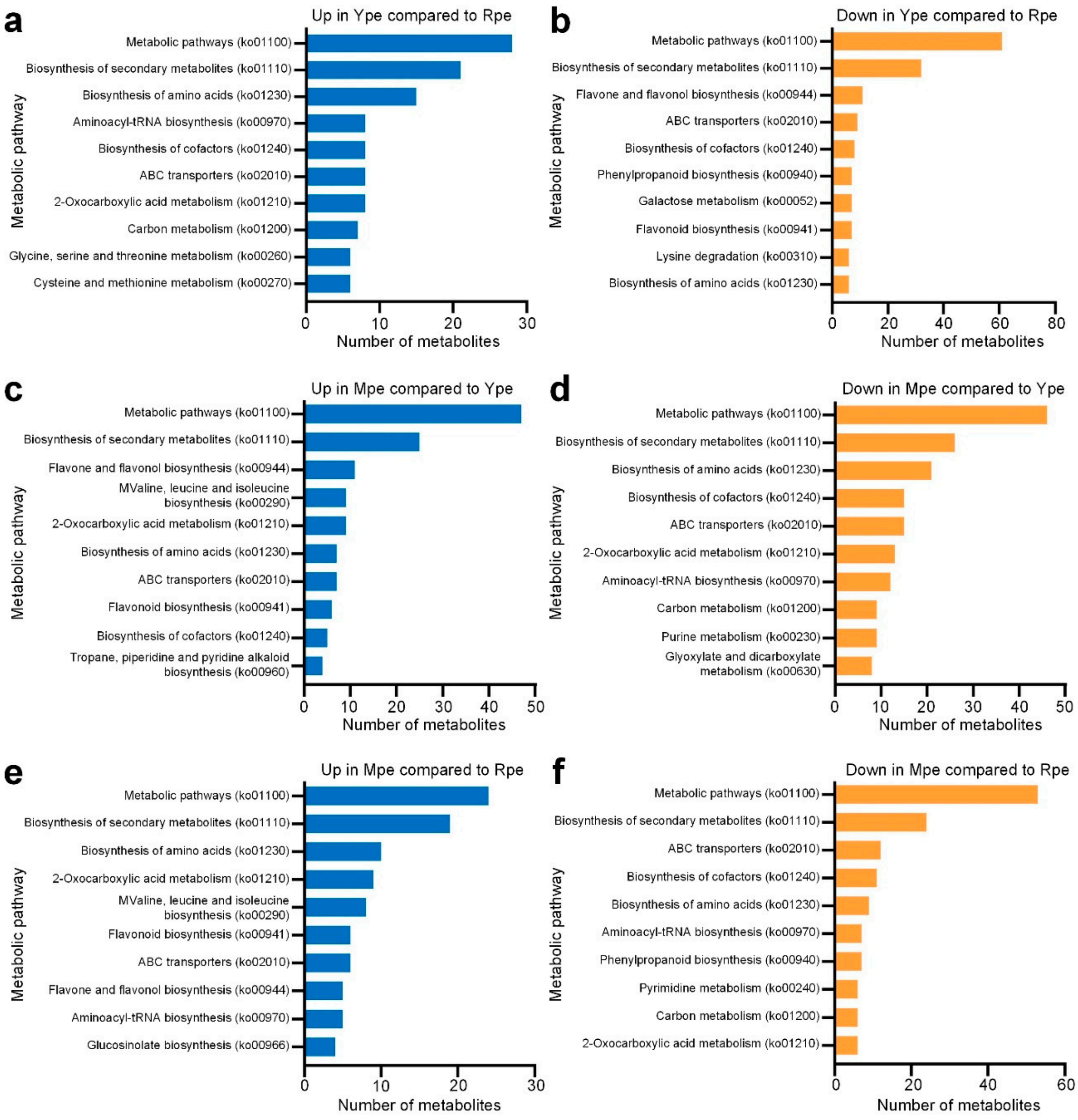

Figure S1. The enriched KEGG pathways of the metabolites with significantly different abundances when comparing Ype to Rpe, Mpe to Ype and Mpe to Rpe, respectively. The x-axis is the number of metabolites enriched in different metabolic pathways. (**a**) Upregulated metabolites in Ype compared to Rpe. (**b**) Downregulated metabolites in Ype compared to Rpe. (**c**) Upregulated metabolites in Mpe compared to Ype. (**d**) Downregulated metabolites in Mpe compared to Ype. (**e**) Upregulated metabolites in Mpe compared to Rpe. (**f**) Downregulated metabolites in Mpe compared to Rpe. The source data are available in Table S41.

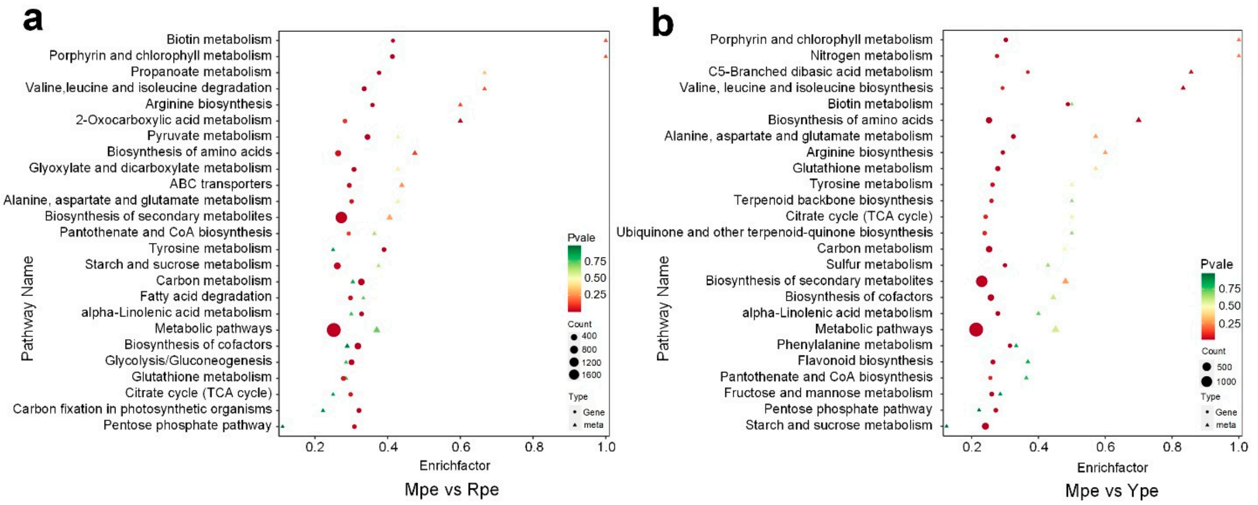

Figure S2. Combined analysis of enriched KEGG pathways of deregulated genes and deregulated metabolites. (**a**) Enriched KEGG pathways when comparing Mpe to Rpe. (**b**) Enriched KEGG pathways when comparing Mpe to Ype.

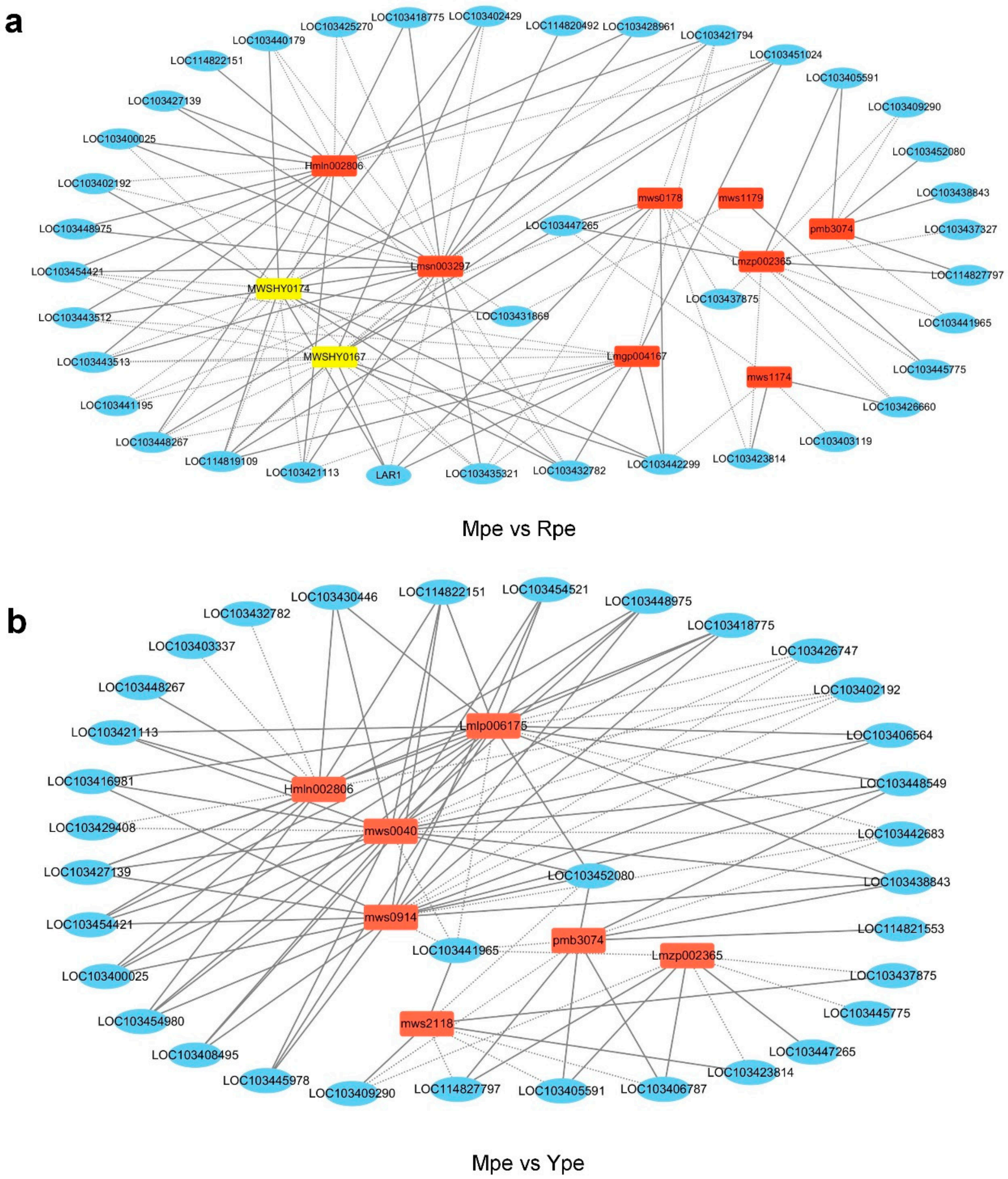

Figure S3. Correlation networks of differentially expressed genes and metabolites that involved in flavonoid biosynthesis pathways. Blue ovals represent genes. Yellow and red rectangles represent metabolites that are upregulated in Ype and Rpe, respectively. Solid and dot lines represent positive and negative correlations, respectively. (**a**) Correlation network for the comparison of Mpe vs Rpe. (**b**) Correlation network for the comparison of Mpe vs Ype. The source data of Part (a) and (b) are available in Table S42 and S43, respectively. Also see Table S2 and Table S15 for the annotations of genes and metabolites, respectively.

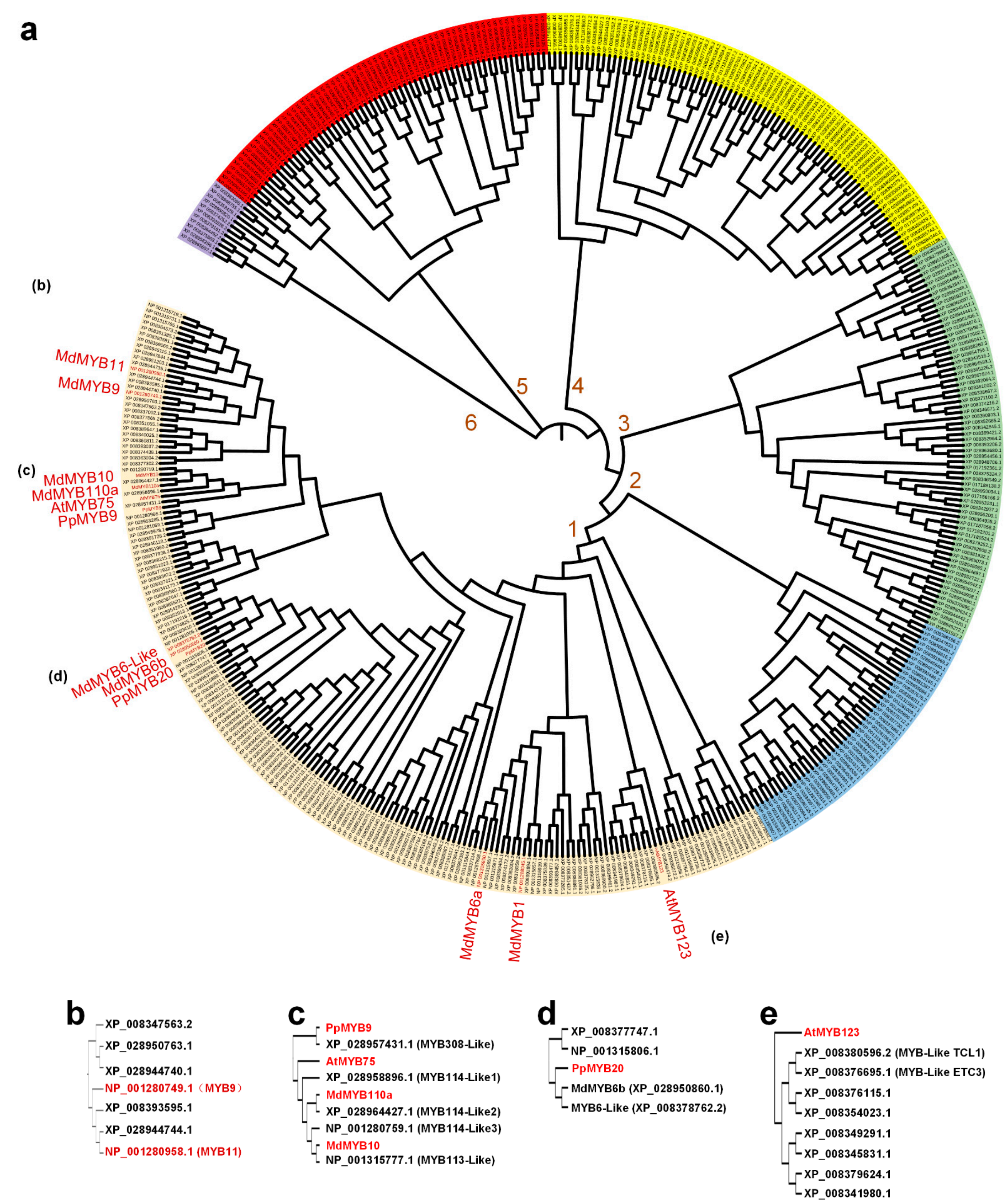

Figure S4. Phylogenetic tree of MYBs in apple and MYBs related to anthocyanin biosynthesis. (**a**) Phylogenetic tree of 442 MYB of apple and 4 MYB of other species. (**b**) - (**e**) Enlarged view of (b) - (e) in Part (a). The protein sequences were used to build the tree. Genes in red represent genes associated with anthocyanin biosynthesis in apple and other species that have been reported. The source data are available in Table S44.

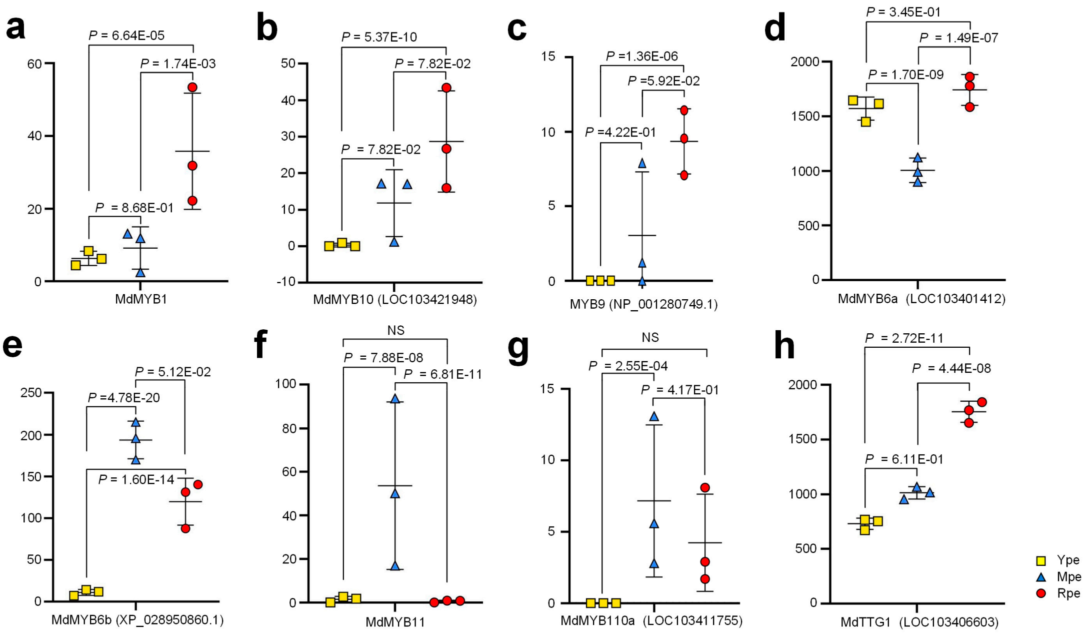

Figure S5. Comparisons of the expression levels of 7 selected genes that played roles in anthocyanin synthesis in the three groups of apple peel samples. (**a**) MdMYB1. (**b**) MdMYB10 (LOC103421948). (**c**) MdMYB9 (NP_001280749.1). (**d**) MdMYB6a (LOC103401412). (**e**) MdMYB6b (XP_028950860.1). (**f**) MdMYB11. (**g**) MdMYB110a (LOC103411755). (**h**) MdTTG1 (LOC103406603). The source data are available in Table S45.

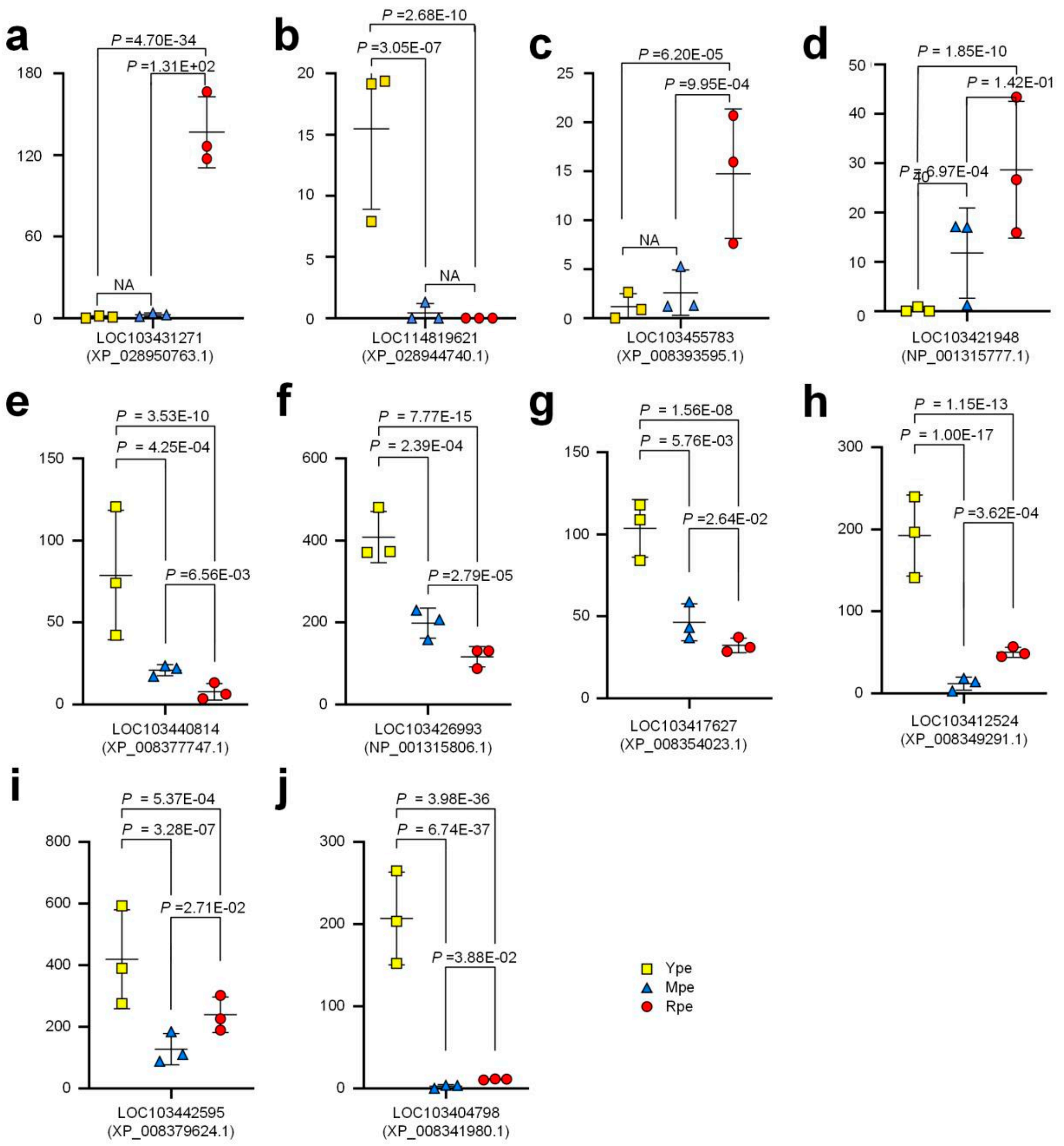

Figure S6. Comparisons of expression levels of 10 MYB genes in the three groups of apple peel samples. (**a**) LOC103431271. (**b**) LOC114819621. (**c**) LOC103455783. (**d**) LOC103421948. (**e**) LOC103440814. (**f**) LOC103426993. (**g**) LOC103417627. (**h**) LOC103412524. (**i**) LOC103442595. (**j**) LOC103404798. The source data are available in Table S46.
